# Hospitalisation of severely mentally ill patients with and without problematic substance use before and during Assertive Community Treatment: an observational cohort study

**DOI:** 10.1186/s12888-016-0826-5

**Published:** 2016-05-04

**Authors:** Hanne Clausen, Torleif Ruud, Sigrun Odden, Jūratė Šaltytė Benth, Kristin Sverdvik Heiervang, Hanne Kilen Stuen, Helen Killaspy, Robert E. Drake, Anne Landheim

**Affiliations:** Department of Research & Development, Mental Health Services, Akershus University Hospital, Lørenskog, Norway; Institute of Clinical Medicine, University of Oslo, Oslo, Norway; National Centre for Dual Diagnosis, Innlandet Hospital Trust, Brumunddal, Norway; HØKH Research Centre, Akershus University Hospital, Lørenskog, Norway; Division of Psychiatry, University College London, London, UK; Dartmouth Psychiatric Research Center, Lebanon, New Hampshire USA; Addiction Research, University of Oslo, Oslo, Norway

**Keywords:** Assertive community treatment, Problematic substance use, Hospitalisation, Inpatient care

## Abstract

**Background:**

Co-occurring substance use increases the risk of hospitalisation in people with severe mental illness, whereas Assertive Community Treatment (ACT) generally reduces hospitalisation in patients with severe mental illness and high inpatient service use. Because the superiority of ACT over standard services amongst patients with problematic substance use is uncertain, the present study examined inpatient service use amongst patients with and without problematic substance use in the 2 years before and the 2 years after they enrolled into ACT teams.

**Methods:**

This naturalistic observational study included 142 patients of 12 different ACT teams throughout Norway. The teams assessed the patients upon enrolment into ACT using clinician-rated and self-reported questionnaires. We obtained hospitalisation data from the Norwegian Patient Register for the 2 years before and the 2 years after enrolment into ACT. We used linear mixed models to assess changes in hospitalisation and to explore associations between problematic substance use and changes in hospitalisation, controlling for socio-demographic and clinical characteristics.

**Results:**

A total of 84 (59 %) participants had problematic substance use upon enrolment into the ACT teams. In the 2 years after ACT enrolment both participants with and without problematic substance use experienced a reduction in total inpatient days. Those with problematic substance use also had fewer involuntary inpatient days. Exploratory analyses suggested that symptom severity and functioning level interacted with problematic substance use to influence change in total inpatient days.

**Conclusion:**

These findings may suggest that ACT teams successfully support people with complex mental health problems in the community, including those with problematic substance use, and thereby contribute to a reduction in inpatient service use.

## Background

Substance use problems are more common amongst patients with schizophrenia than in the general population [1] with reported lifetime prevalence ranging from 47 to 60 % [1–3] and current prevalence ranging from 27 to 41 % [2, 4]. Substance use problems amongst people with schizophrenia also increase the risk of many negative outcomes, including increased hospital readmissions [5, 6], number of inpatient days [7], and involuntary admissions [8].

Assertive Community Treatment (ACT) is an intensive, multidisciplinary, community-based mental health service model that reduces hospitalisation amongst people with severe mental illnesses, such as schizophrenia, that are high users of inpatient care [9]. Many also suffer comorbidities, do not engage successfully with standard mental health services [10], and experience recurrent cycles of relapse, hospital readmissions [10, 11], and high use of inpatient services [9, 12, 13]. The ACT approach provides more flexible and intensive support than generic mental health services, delivering evidence-based, individually-tailored interventions in the community [10, 14]. The prevalence of current substance abuse in ACT populations ranges from 49 to 72 % [15–18], higher than other mental health outpatient groups.

Although ACT is generally superior to standard community-based services in reducing hospitalisation, the evidence amongst patients with co-occurring substance misuse problems is equivocal [19–21]. Few studies have compared hospitalisation amongst patients with and without co-occurring substance misuse problems before and after they engaged with ACT. One previous study that explored associations between patient characteristics and changes in hospitalisation, found that changes in total and involuntary inpatient days were not associated with the severity of alcohol or drug use problems. [22] Nevertheless, because substance use increases the risk of hospitalisation, patients with co-occurring substance misuse problems are likely to be higher users of inpatient services. We therefore hypothesized that ACT would have a greater impact on hospitalisation amongst this group, even though ACT has not been proven to effectively reduce substance use [19].

### Aims and research questions

The aims of this study were to compare inpatient service use (new admissions, total inpatient days, and involuntary inpatient days) amongst ACT patients with and without problematic substance use and to explore associations between changes in inpatient service use and patient characteristics, including problematic substance use.

Our research questions were: First, are there differences in inpatient service use amongst patients with and without problematic substance use during the 2 years before and the 2 years after ACT enrolment? Second, is problematic substance use associated with changes in hospitalisation when adjusted for patient characteristics?

## Methods

### Design

We used a naturalistic observational study on ACT in Norway. For 142 patients of 12 Norwegian ACT teams, we combined cross-sectional socio-demographic and clinical data from enrolment into ACT and longitudinal hospitalisation data in the 2 years before and the 2 years after enrolment. Due to the nature of the funding and the implementation of the ACT model in Norway, we could not conduct a randomized trial.

### Recruitment and sample

Between 1999 and 2008 a national program took place in Norway to improve mental health services. However, the evaluation of the program concluded that, despite major investments, expansion and reorganisations, the services were lacking continuity, they were fragmented, and approximately 4000 people with severe mental illness were not well engaged with services despite their need for treatment and follow-up [23]. Subsequently, in 2009, the National Health Authorities decided to fund the implementation of ACT teams across Norway to improve services for people with severe mental illness who suffered comorbidities such as substance misuse and needed more comprehensive services. A history of high inpatient service use was not mandatory for being taken on by the ACT teams because the aim was to reach people who were not well engaged with services. This could potentially include patients who had not been frequently admitted to hospital.

Between December 2009 and February 2011, 12 ACT teams started up across the country. Patient inclusion criteria included: 18 years or older, severe mental illness (schizophrenia, schizoaffective, other psychotic disorder, bipolar affective disorder), impaired level of functioning, and need for long-term, comprehensive follow-up by mental health and social welfare services.

Patients with co-occurring substance misuse were included if this was not the primary diagnosis.

A severe mental illness was diagnosed by referring agencies and was based on International Statistical Classification of Diseases and Related Health Problems 10th Revision (ICD-10) criteria [24] for 69 participants (49 %); upon the “Mini International Neuropsychiatric Interview Plus” (MINI Plus) [25], or the “Structured Clinical Interview for DSM-IV Axis I Disorders” (SCID­I) [26], or other non-specified diagnostic instrument for 6 participants (4 %) while it was unknown how 27 participants (19 %) were diagnosed. Data were missing for 40 participants (28 %).

The use of the Global Assessment of Functioning (GAF) scale is mandatory in specialised mental health care in Norway but not in primary care. The referral agencies therefore assessed the level of functioning based on clinical evaluation or on the GAF scale [27, 28].

For the present study we limited inclusion to the ACT teams’ first year of operation. A total of 337 patients enrolled into the 12 teams and all patients were invited to participate in the study; 202 (60 %) gave written informed consent to participate after the teams had explained the procedures; and 142 participants (42 %) received ACT services for at least 2 years and were thus eligible for this study.

All 142 participants remained in contact with the teams during the 2 year follow-up period. A total of 12 participants (8 %) were admitted to inpatient substance use treatment in the 2 years before and/or after being taken on by the ACT teams. While three participants only were admitted in the 2 years before, seven participants were only admitted during ACT follow-up and two participants were admitted both before and during ACT follow-up. The mean number of inpatients days spent in substance abuse treatment in the 2 years before ACT was 17.4 days (SD 11.1) and median 13 days (min-max: 7–34 days). The mean number of inpatient days spent in substance abuse treatment during ACT follow-up was 39.3 days (SD 53.0) and median 11.0 days (min-max: 1–133 days). We have no data on periods of incarceration.

Participants and non-participants did not differ in age, gender, diagnosis of severe mental illness, or number of people being subject to involuntary outpatient treatment. Participants did, however, have less severe symptoms (mean score ± Standard Deviation [SD] on Global Assessment of Functioning – Symptom Scale [GAF-S], 41.4 ± 10.2 versus 38.8 ± 10.0, *p* = 0.028) and better functioning (mean score ± SD Global Assessment of Functioning – Function Scale [GAF-F], 39.7 ± 8.3 versus 37.6 ± 8.9, *p* = 0.036). Upon enrolment into ACT, fewer participants had problematic substance use (*n* = 83 versus 128, 59 versus 70 %, *p* = 0.034) compared to non-participants.

Most participants were male (*n* = 94, 67 %), and of Norwegian origin (*n* = 114, 84 %). They had a mean age of 39.8 ± 10.6 years. Most were single (*n* = 106, 75 %), living alone (*n* = 91, 65 %), and unemployed (*n* = 118, 83 %). Few had completed higher education (*n* = 12, 9 %). Almost all had a severe mental illness (according to the ICD-10 criteria, *n* = 124, 94 %) such as schizophrenia (F20-29, *n* = 115, 87 %) or bipolar disorder (F31, *n* = 9, 7 %). The mean age of illness onset was 25.9 ± 8.7 years. Overall, participants experienced severe symptoms (GAF-S 41.4 ± 10.2) and poor functioning (GAF-F 39.7 ± 8.3) at the point of enrolment (these scales are described in more detail below).

### Measures

Problematic substance abuse was assessed using two self-reported questionnaires, The Alcohol Use Disorder Identification Test (AUDIT) [29] and the Drug Use Disorder Identification Test (DUDIT) [30], and two clinician-rated questionnaires, the Alcohol Use Scale (AUS) [31] and the Drug Use Scale (DUS) [32]. The AUDIT comprises 10 items with total score from 0 to 40 and the DUDIT comprises 11 items with total score from 0 to 44. Scores above specific cut-offs (AUDIT: men 8, women 6; DUDIT: men 6, women 2) indicate problematic substance use and higher scores indicate greater severity. The AUS and the DUS are 5-point scales with scores from 1 (no use) to 5 (severe dependence), with score 3 or higher indicating problematic substance use.

The ACT team clinicians also collected socio-demographic data using a form developed by the research group (*life situation and health-questionnaire*), and patients’ global symptom and functioning levels using the Global Assessment of Functioning (GAF) scale [27], split version (symptoms scale [GAF-S] and functioning scale [GAF-F]) [28]. The GAF scales range from 0 to 100, and higher scores indicate less severe symptoms and better functioning. The expanded version of the Brief Psychiatric Rating Scale (BPRS) [33, 34] was used to assess the frequency and severity of psychiatric symptoms. The BPRS comprises 24 items, yielding four factors (positive symptoms, negative symptoms, agitation mania, and anxiety/depressive symptoms) [35]. Each item is rated from 1 (not present) to 7 (extremely severe). Everyday functioning was measured with the revised version of the Practical and Social Functioning Scale (PSF) [36]. PSF-revised comprises 32 items with a mean total score ranging from 0 to 8. Higher scores indicate better functioning.

### Procedures

We obtained data from the Norwegian Patient Register on inpatient service use in mental health hospitals for the 142 patients in the 2 years before and the 2 years after enrolment into ACT. We used data from both clinician-rated and self-reported questionnaires. The ACT teams collected socio-demographic and clinical data when patients enrolled into teams through interviews with patients, care givers, professionals, and from direct observations and case-note reviews. Patients responded to the self-reported questionnaires (the AUDIT and the DUDIT) alone or together with a team member at enrolment onto the teams. The teams repeated the AUS and the DUS after 2 years with ACT while the participants in the study completed the AUDIT and the DUDIT after 2 years of ACT follow-up.

### Fidelity of Norwegian ACT teams

The Norwegian teams’ fidelity to the ACT model was assessed using the Tool for Measurement of Assertive Community Treatment (TMACT) [14] 12 and 30 months after establishment. The mean TMACT scores at 12 months ranged from 2.7 to 3.7, indicating low to moderate fidelity and at 30 months the scores ranged from 3.1 to 4.1, indicating moderate to high fidelity. The key principles of ACT, mainly measured on the subscales organization & structure, core team members, and core practices, represent the greatest differences with Norwegian standard mental health services. The ratings on these subscales showed moderate to high fidelity at both 12 and 30 months. Substance abuse specialist was present in 11 teams at 12 and 30 months fidelity evaluation. The mean TMACT scores on the five subscales relating to substance abuse specialist and Integrated Dual Disorder Treatment (IDDT) showed moderate to high fidelity. However, the scores on the different items showed large variations between teams (scores ranged 1–5, indicating none to full implementation).

### Statistical analysis

We assessed differences in demographic and clinical characteristics between groups by Fisher’s exact test for dichotomous variables, Chi-square test for categorical variables, Student’s *T*-test for symmetrically distributed continuous variables, and Mann–Whitney *U* test for skewed continuous variables.

Three dependent variables assessed the change in hospitalisation; *new admissions, total inpatient days and involuntary inpatient days*. We defined these three dependent variables as the difference between the number 2 years before and the number 2 years after enrolment into ACT.

We analysed the difference between participants with and without problematic substance use in the three dependent variables by estimating linear mixed models, one for each variable. The models contained fixed effect for each patient group (with and without problematic substance use). Random effects for intercepts were included into the models to adjust for possible cluster effect due to intra-ACT correlations.

In the *exploratory* multivariate linear mixed models, we adjusted the associations between problematic substance use [Y/N] and the three dependent variables for demographic (age, gender) and clinical factors (involuntary outpatient treatment [Y/N], the four BPRS subscales, GAF-S, GAF-F, and PSF). In the same exploratory analyses, we also assessed interactions between the problematic substance use variable and demographic and clinical characteristics in all three models. We used Akaike’s Information Criteria [37] (the smaller the better) in model reduction. We applied standard residual diagnostic tests to assess the assumption of linear mixed models. The residuals were somewhat skewed, therefore we generated bootstrap based inference as well. However, as the differences were negligible, the results from the linear mixed model were presented. We considered these exploratory analyses as hypothesis-generating and not hypothesis-testing; therefore we did not correct for multiple tests.

We imputed missing values on PSF items (*n* = 14, 0.3 % of cases) by generating the empirical distribution for each item and drawing a random number from that distribution to replace the missing value. The process was repeated until all missing values were imputed. The GAF-S and GAF-F scores were close to normally distributed, and missing values (both *n* = 4, 2.8 % of cases) were therefore imputed by drawing a random number from the corresponding normal distribution. The BPRS was completed for 98.6 % of the participants and thus we imputed no scores. As the number of imputed values was low, no sensitivity analysis was performed.

We used the Statistical Analysis System version 9.3 (SAS Institute, Cary, NC USA) to estimate linear mixed models and the Statistical Package for Social Science version 22 (SPSS, Chicago, IL USA) for other statistical analyses. All tests were two-sided, considering *P*-values below 0.05 as statistically significant.

## Results

### Classification and characteristics of participants with and without problematic substance use

We based classification of problematic substance use primarily on the AUDIT and DUDIT scores. Seventy-two participants (51 %) had a score above cut-off on one or both scales. The mean AUDIT and DUDIT scores ± SD for participants with scores above cut-off indicated severe problems (AUDIT 17.1 ± 7.6 and DUDIT 21.0 ± 10.3).

For participants who had not completed the AUDIT and DUDIT (*n* = 12, 8 %) or who had a score below cut-off (*n* = 58, 41 %), we added the clinician-rated AUS and DUS. For nine participants the clinicians gave a score of 3 or higher on the AUS and/or the DUS, and we classified these participants as having problematic substance use. In addition, we identified three participants with missing AUS and DUS as having problematic substance use based on the clinician-rated assessment of substance abuse in the life situation and health-questionnaire.

Thus, 84 (59 %) participants had problematic substance use, while 58 (41 %) did not. The most commonly used substances were alcohol (*n* = 54, 74 %), amphetamine (*n* = 34, 54 %) and cannabis (*n* = 30, 52 %).

After 2 years with the ACT teams, 78 patients (93 %) still had problematic substance use. Four (7 %) of the 58 participants who were originally classified as not having a problem met the criteria for problematic substance use on follow-up, while six of the 84 participants (7 %) who had problematic substance use upon ACT enrolment no longer met the criteria after 2 years.

The mean scores ± SD on the AUDIT (16.2 ± 7.7) and the DUDIT (22.8 ± 10.0) for those who scored above cut-off again indicated severely problematic substance use at 2 years follow-up.

Table 1 presents the characteristics of each group upon ACT enrolment. Participants with problematic substance use were more likely to be of Norwegian origin, under involuntary outpatient treatment, and to have a lower level of educational achievement than participants without problematic substance use. They also had more severe psychiatric symptoms, in particular manic/agitated symptoms, and poorer functioning than participants without problematic substance use.

**Table 1:** Socio-demographic and clinical characteristics of participants with and without problematic substance use on ACT enrolment

Socio-demographic characteristics:	Non-substance group (*N* = 58)		Substance group (*N* = 84)		
	*N*	%	*N*	%	*P*-value
Sex (male)	34	59.6	60	71.4	0.151^a^
Age, *mean (SD)*	41.7 (11.7)		38.4 (9.6)		0.068^c^
Ethnicity					0.001^a^
Norwegian	38	70.4	76	92.7	
Marital status					0.056^b^
Unmarried	38	65.5	68	81.0	
Married/cohabitant	5	8.6	7	8.3	
Divorced	15	25.9	9	10.7	
Education					0.003^b^
Completed primary school	29	55.8	47	58.8	
Completed upper secondary school	13	25.0	31	38.8	
Completed higher education	10	19.2	2	2.5	
Employment status					0.291^b^
Unemployed	45	77.6	73	86.9	
Competitive job/study	5	8.6	3	3.6	
Other	8	13.8	8	9.5	
Living situation					0.625^b^
Alone	38	65.5	53	63.9	
With family	14	24.1	17	20.5	
Staffed housing/supported housing/Institutions (hospital, prison, hospice)/Homeless/unstable living situation	6	10.3	13	15.7	
Clinical characteristics:					
Diagnosis					0.710^a^
Severe mental illness (yes)	47	95.9	77	92.8	
Community treatment order (yes)	13	22.4	38	45.8	0.005^a^
Age of onset psychiatric illness, *mean (SD)*	27.3	9.4	24.8	8.1	0.135^d^
Psychiatric symptoms, *mean (SD)*					
BPRS mean total score, *mean (SD)*	2.24	0.66	2.60	0.86	0.015^d^
BPRS positive symptoms, *mean (SD)*	2.23	1.14	2.65	1.34	0.075^d^
BPRS negative symptoms, *mean (SD)*	2.59	1.18	2.43	1.14	0.432^d^
BPRS agitation mania, *mean (SD)*	1.78	0.77	2.42	1.19	0.001^d^
BPRS anxiety/depressive symptoms, *mean (SD)*	2.63	1.10	2.77	0.95	0.425^c^
Global level of functioning – symptom scale (GAF-S), *mean (SD)*	43.6	10.6	39.8	9.8	0.032^c^
Global level of functioning – functioning scale (GAF-F), *mean (SD)*	40.8	8.6	38.9	8.1	0.161^c^
Level of functioning (PSF), *mean (SD)*	4.63	1.62	4.05	1.50	0.033^c^

^a^Fischer’s Exact Test

^b^Chi-square

^c^Student’s *T*-test

^d^Mann–Whitney *U* Test

### Changes in hospitalisation

Of the 142 participants in our study, 128 (90 %) were admitted to mental health hospital in the 2 years before and/or the 2 years after being taken on by the ACT teams. A total of 14 participants (10 %) were not admitted at all. Of these 14 participants, nine (64 %) did have problematic substance use while five (36 %) did not. Table 2 shows the mean number of new admissions, mean total inpatient days and mean involuntary inpatient days in the 2 years before and the 2 years after ACT enrolment for all participants with and without problematic substance use. According to the linear mixed models unadjusted for patient characteristics, the mean number of new admissions did not change after ACT enrolment in either group, but both groups experienced reduction in total inpatient days. Patients with problematic substance use also had fewer involuntary inpatient days after being taken on by ACT.

**Table 2:** Hospitalisation during two years before and after ACT: participants with and without problematic substance use

		Before taken on by ACT		After taken on by ACT		Change before-after taken on by ACT		
		Mean	SD	Mean	SD	Mean^a^	95 % confidence interval	*P*-value^b^
New admissions	Non-problematic substance use^c^	2.79	3.06	2.78	5.07	0.05	−1.31 to 1.40	0.945
	Problematic substance use^d^	3.71	4.48	3.26	4.48	0.45	−0.68 to 1.57	0.436
Total inpatient days	Non-problematic substance use^c^	106.12	133.83	50.55	57.18	58.24	7.83 to 108.64	0.024
	Problematic substance use^d^	131.15	167.51	69.01	88.54	64.09	21.90 to 106.28	0.003
Involuntary inpatient days	Non-problematic substance use^c^	51.53	116.51	20.78	40.07	29.96	−14.92 to 74.83	0.191
	Problematic substance use^d^	101.05	163.09	47.57	75.99	55.69	19.16 to 92.22	0.003

^a^Positive means indicate mean reduction in outcome after being taken on by ACT compared to before while negative means indicate mean increase in outcome

^b^Analyses of change using linear mixed models, unadjusted model

^c^Non-substance group *n* = 58 (41 %), ^d^ Substance group *n* = 84 (59 %)

### Associations between problematic substance use and changes in hospitalisation

We found only small differences between the ACT teams regarding changes in all hospital outcomes, as indicated by the low intra-class correlation coefficients (new admissions 2.7 %, total inpatient days 3.7 %, and involuntary inpatient days 1.4 %), but we adjusted all models for cluster effects.

No significant interactions occurred between problematic substance use and the adjustment variables (demographic characteristics [age, gender] or clinical characteristics [BPRS four factors, GAF-S, GAF-F and PSF]).

The multivariate exploratory linear mixed models showed no associations between problematic substance use and changes in the number of new admissions or involuntary inpatient days but significant associations with change in total inpatient days emerged (Table 3). Symptom severity and functioning levels influenced these associations.

**Table 3:** Linear mixed models: Associations between problematic substance use and changes in hospitalisation (*n* = 128)

Variables	New admissions		Total inpatient days		Involuntary inpatient days	
	Regression coefficient (SE)	*p*-value	Regression coefficient (SE)	*p*-value	Regression coefficient (SE)	*p*-value
Problematic substance use (Y/N)	0.35 (0.91)	0.698	−113.00 (151.19)	0.456	56.30 (30.21)	0.065
BPRS Positive symptoms	0.35 (0.45)	0.439	−11.01 (16.23)	0.499	−13.93 (14.78)	0.348
BPRS negative symptoms	−0.45 (0.38)	0.230	19.83 (13.97)	0.159	17.70 (12.71)	0.166
BPRS agitation mania	0.56 (0.51)	0.277	−34.80 (19.49)	0.077	−35.55 (17.35)	0.043
BPRS anxiety/depressive symptoms	−0.88 (0.43)	0.042	−20.58 (15.24)	0.180	−19.15 (14.18)	0.180
GAF-S	0.16 (0.07)	0.027	3.46 (3.38)	0.308	−2.45 (2.36)	0.301
GAF-F	−0.12 (0.08)	0.151	−9.18 (4.38)	0.037	−0.68 (2.80)	0.809
PSF	–	–	15.02 (11.40)	0.191	8.68 (10.16)	0.395
Age	0.01 (0.04)	0.819	−1.95 (1.56)	0.214	−0.46 (1.45)	0.754
Gender	−0.58 (0.89)	0.514	51.78 (32.46)	0.114	53.40 (29.41)	0.072
Problematic substance use*GAF-S			−8.25 (4.52)	0.071*		
Problematic substance use*GAF-F			12.31 (5.77)	0.035*		

*P-values below 0.10 were considered significant for interactions

**Fig. 1:**
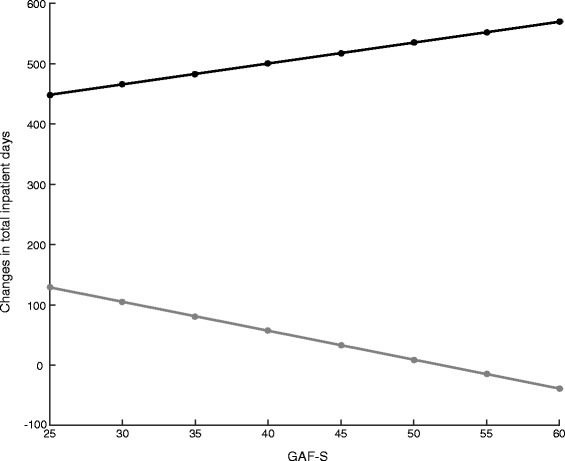
Association between changes in total inpatient days and level of symptoms for both groups: *Black line*: Participants without problematic substance use. *Grey line*: Participants with problematic substance use

Less severe symptoms were associated with greater reduction in total inpatient days amongst participants without problematic substance use, but no association occurred between symptom severity and changes in total inpatient days amongst participants with problematic substance use (see Fig. 1).

**Fig. 2:**
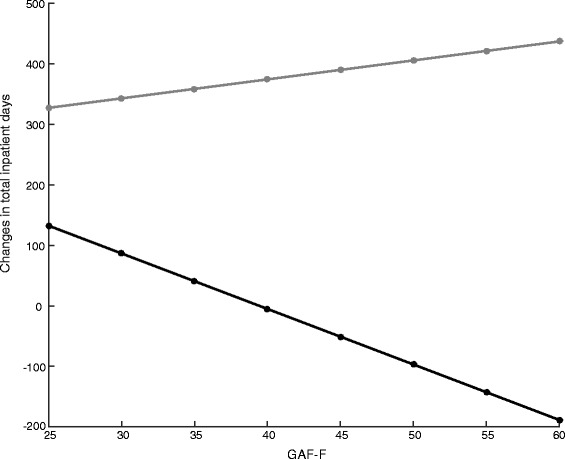
Association between changes in total inpatient days and level of functioning for both groups: *Grey line*: Participants with problematic substance use. *Black line*: Participants without problematic substance use

Participants with less seriously impaired functioning (GAF-F score 45 or above) and problematic substance use experienced a reduction in total inpatient days while participants without problematic substance use with similar functioning level accrued more inpatient days in the 2 years with ACT compared to the 2 years before (see Fig. 2).

Additionally, amongst participants with more severely impaired functioning (GAF-F score up to 50) and problematic substance use, better functioning was associated with greater reduction in total inpatient days. This association was not found amongst participants with problematic substance use and less severely impaired functioning or amongst participants without problematic substance use.

We explored the linear mixed models further by adjusting for the change in status of 10 participants regarding their problematic substance use (six participants who had problematic substance use upon ACT enrolment no longer met the criteria after 2 years and four who did not have problematic substance use originally had developed these at the 2 year assessment point). The results remained unchanged.

## Discussion

A total of 84 (59 %) participants had problematic substance use when they enrolled into the ACT teams and after 2 years with ACT, 78 (93 %) participants still had ongoing, severe substance use.

We found no changes in the number of new admissions after the participants enrolled into the ACT teams, but total inpatient days decreased. Participants with problematic substance use also had fewer involuntary inpatient days during ACT follow-up.

Exploratory analyses showed that changes in total inpatient days differed for participants with compared to participants without problematic substance use, and symptom severity and functioning level upon ACT enrolment influenced these changes.

### Changes in hospitalisation

Our results confirm earlier reports in that total inpatient days were reduced during ACT, but without significant reduction in the number of admissions [9]. The reduction in total and involuntary inpatient days amongst participants with problematic substance use occurred despite on-going substance use. This may be explained by their high level of inpatient service use before being taken on by the ACT teams. [9] Our findings indicate that ACT successfully reduces inpatient service use amongst patients with and patients without problematic substance use.

Changes in inpatient service use could also be influenced by temporal changes in national policies and bed availability. This threat to validity emerges particularly in uncontrolled pre-post studies such as ours. From 2009 to 2013, a reduction of only 13 % in inpatient mental health beds and 15 % in total inpatient days occurred in Norway [38], and fluctuations in the number of involuntary inpatient days were minor [39]. Additionally, national data from 2009 [40] to 2013 [38] showed that high users of inpatient services, the majority of whom suffer severe mental illness like schizophrenia, spent an average of 75–83 days in hospital per year. This is similar to the level of total inpatient days per year in the 2 years before ACT in our study but almost twice as high as the number of total inpatient days per year during the ACT follow-up. This suggests that national changes in policies and bed availability cannot fully explain the reductions in our study and that the changes do not represent temporal effects. However, we cannot exclude that the changes observed in this study are regression to the mean.

Qualitative data have suggested that the ACT teams identified participants in an early state of relapse, thereby avoiding severe deteriorations that might have required long-term admissions [41].

Participants may also have been discharged earlier because of the availability of support and services from high intensity ACT teams.

### Associations between problematic substance use and changes in hospitalisation

Exploratory linear mixed models showed no associations between problematic substance use and changes in the number of new admissions, or between problematic substance use and change in involuntary inpatient days despite a significant reduction amongst participants with problematic substance use and not amongst participants without problematic substance use. However, total inpatient days changed differently for participants with problematic substance use compared to those without in the sense that symptom severity and functioning level influences these changes.

These results were from exploratory analyses performed in a small sample, aiming to generate hypothesis. This aspect of our study may be under-powered and need replication before conclusions can be drawn.

### Strengths and limitations

Strengths of our study included: data from 12 ACT teams operating in both urban and rural areas across Norway; instruments with good psychometric properties; and 4 years of longitudinal data. Weaknesses included: the observational design, which weakens causal interpretations; the high rate of non-participation that could lead to an overestimation of change in hospitalisation in one or both groups because fewer patients with more severe illness participated.; potential errors in the data from the Norwegian Patient Register; all teams were newly established which may have had positive effects in that the ACT staff were motivated, enthusiastic and had (at least in the start-up phase) a low patient:staff-ratio. The negative effects may be that they implemented an unfamiliar model (to the Norwegian health system), did not have all necessary resources in place and lacked skills and training in providing evidence based treatment. Further limitations were the use of clinician-rated instruments and the large number of clinicians involved in the assessments; and the presumed accuracy of our multi-method diagnosis of problematic substance use which may have caused an under- or over representation of people with problematic substance use and thereby influenced an under- or overestimation of change in hospitalisation.

## Conclusion

This study found that participants with and without problematic substance use had significant reductions in inpatient days during the ACT follow-up. In addition, those with problematic substance use also had fewer involuntary inpatient days, despite on-going problematic substance use. These findings may suggest that ACT teams successfully support people with complex mental health problems in the community, including those with problematic substance use, and thereby contribute to a reduction in inpatient service use.

### Ethics and consent to participate

The Regional Committee for Medical and Health Research Ethics Health Region South-East approved the study (ID: 2010/1196a) and all participants included in this paper have given written informed consent to participate after the ACT teams explained the procedure to them.

### Consent to publish

Not applicable.

### Availability of data and materials

The written consent from the participants does not allow for distribution of the data file to others than the research group that conducted the study. Other researchers that want access to the data may contact the principal investigator (TR), who will answer whether the requested data may be made available in a form that does not violate the written consent from the participants.

## Abbreviations

ACT: Assertive Community Treatment
AUDIT: Alcohol Use Disorder Identification Scale
AUS: Alcohol Use Scale
BPRS: Brief Psychiatric Rating Scale
DUDIT: Drug Use Disorder Identification Scale
DUS: Drug Use Scale
GAF: Global Assessment of Functioning (GAF-S: symptom scale, GAF-F: function scale)
ICD-10: International Statistical Classification of Diseases and Related Health Problems 10th Revision
IDDT: Integrated Dual Disorder Treatment
MINI Plus: Mini International Neuropsychiatric Interview Plus
PSF: Practical and Social Functioning
SAS: Statistical Analysis System
SCID-I: Structured Clinical Interview for DSM-IV Axis I Disorders
SD: standard deviation
SPSS: Statistical Package for Social Science
TMACT: Tool for Measurement of Assertive Community Treatment
